# Reply to “Comments on the Editor Re: Shi, Zumin, et al. High Chili Intake and Cognitive Function among 4582 Adults: An Open Cohort Study over 15 Years. Nutrients 11.5 (2019): 1183.”

**DOI:** 10.3390/nu11122882

**Published:** 2019-11-26

**Authors:** Zumin Shi, Tahra El-Obeid, Malcolm Riley, Ming Li, Amanda Page, Jianghong Liu

**Affiliations:** 1 Human Nutrition Department, College of Health Sciences, QU Health, Qatar University, Doha 2713, Qatar; tahra.e@qu.edu.qa; 2 Commonwealth Scientific and Industrial Research Organisation (CSIRO), P.O. Box 10041, Adelaide, SA 5000, Australia; malcolm.riley@csiro.au; 3 Centre for Population Health Research, Division of Health Sciences, University of South Australia, Adelaide, SA 5000, Australia; Ming.Li@unisa.edu.au; 4 Adelaide Medical School, University of Adelaide, Adelaide, SA 5000, Australia; amanda.page@adelaide.edu.au; 5 University of Pennsylvania School of Nursing, Philadelphia, PA 19104, USA; jhliu@nursing.upenn.edu

We would like to thank Drs. Wang and Wu [1] for their interest in and comments on our recent paper [2]. The following is our response to their specific concerns. 

First, the authors commented on the types of chili peppers analyzed in our study. We did not include sweet capsicum and black pepper in the analysis as they do not contain high levels of capsaicin, the active component of chili. In our survey, we did not differentiate fresh from dry chili, and this limitation has already been acknowledged. On the other hand, dry chili is usually used as a spice in Chinese cuisine, and the consumption of dry chili is relatively lower than fresh chili. Therefore, it can be assumed that misclassification of chili consumption levels is random and will not affect our findings on the association between total chili consumption and cognition.

Second, Yang Y and Wu D commented about the dietary assessment method in our study. Our study used the combination of food weighing and 24 h food recording in the household setting. This method is widely used in population nutrition studies in China (e.g., China National Nutrition Survey [3]) and has been validated for energy intake [4]. As previously reported, the correlation coefficient between the reported energy intake and total energy expenditure determined by the doubly labelled water method was 0.56 (*p* < 0.01) for men and 0.60 (*p* < 0.01) for women [4]. Although sharing dishes is common in the Chinese dining culture, estimation of the approximate consumption proportion within the household is currently the most reasonable method to quantify the consumption. Based on the individual consumption proportion and the total weighed chili consumption, we calculated the individual chili intake.

Third, the authors questioned the validity of the outcome measurement in regards to using composite scores of memories, counting back and subtraction scores, especially among those with low education. However, the face-to-face cognitive screen test is widely used in epidemiological studies. Among participants attending the first cognitive function test, 85.3% of those with a low education attained a full score of the subtraction test. The main difference of the cognition score was from the memory test. In the memory test, the ten words used were mainly related to daily living (i.e., house, wood, cat, table, night, needle, steamed bread, door, bridge, and bed) and less likely to be affected by education. Furthermore, our findings are less likely confounded by age. Among those with a global cognitive score below 7 (18.0% among those attending the first cognitive function test), the mean age of those with a high intake of chili, self-reported memory decline or poor memory was lower than non-consumers of chili (Figure 1). This might suggest that high chili consumption is associated with cognitive impairment at a younger age. 

Fourth, the authors were concerned about the significant loss in the follow-up sample and the exclusion of those who attended only one survey. We agree that loss of follow-up is a limitation in the study. However, the main reason for the loss of follow-up was due to the rapid economic development in China and migration, but not cognition related diseases. In our analysis, we used a stepwise approach to examine the association between chili intake and cognition. We included those that participated in one survey in most of our multivariable models. The exclusion of those who only participated in one survey did not change the finding. 

The fifth comment concerned the lack of adjustment for chronic diseases and medication use. As chili consumption is inversely associated with obesity and hypertension, adjusting for chronic conditions may lead to over adjustment. However, adjustment for hypertension, obesity, diabetes, and stroke did not change the association between chili intake and cognitive function. We agree with the authors that the lack of adjustment of medication is a limitation. 

In conclusion, the study provided the most comprehensive dietary intake data in population studies to assess the association between chili intake and cognition, suggesting that high chili consumption might lead to early onset of cognitive impairment. Future studies should consider differentiating between fresh and dry chili intake, using more objective and multidimensional cognitive assessments, as well as controlling for more confounders to further confirm the relationship between chili intake and cognition.

## Figures and Tables

**Figure 1:**
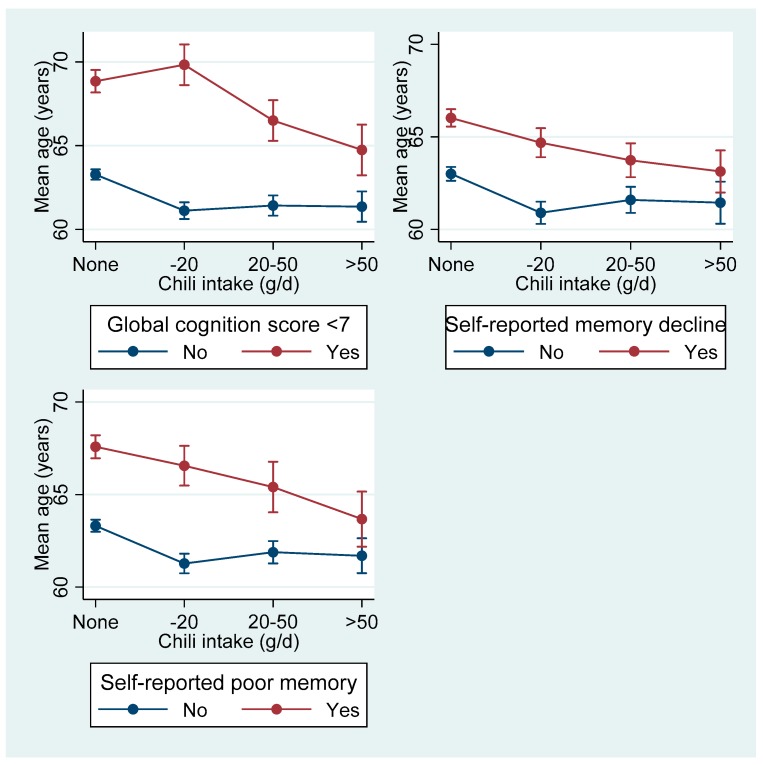
Mean age (95% CI) of participants attending the first cognitive function test by chili intake and cognitive function status.
